# Evaluation of the Training in Early Detection for Early Intervention (TEDEI) e-learning course using Kirkpatrick’s method

**DOI:** 10.1186/s12909-023-04113-7

**Published:** 2023-02-27

**Authors:** Eleanor Officer, Maisie Johnson, Jessica Blickwedel, Ashley Reynolds, Rachel Pearse, Janice Pearse, Anna Purna Basu

**Affiliations:** 1 School of Psychology, Newcastle University, Newcastle upon Tyne, England, UK; 2 Northern Foundation School, Newcastle upon Tyne, England, UK; 3 Faculty of Medical Sciences, Newcastle University, Newcastle upon Tyne, England, UK; 4 North East and North Cumbria GP Training Programme, Newcastle upon Tyne, England, UK; 5 Population Health Sciences Institute, Newcastle University, Newcastle upon Tyne, England, UK; 6 Therapy Services, Newcastle Upon Tyne Hospitals NHS Foundation Trust, Newcastle upon Tyne, England, UK; 7 Paediatric Neurology, Newcastle upon Tyne Hospitals NHS Foundation Trust, Newcastle upon Tyne, England, UK

**Keywords:** E-learning, Cerebral palsy, Assessment, Infant, Healthcare practitioners, Tele-health

## Abstract

**Background:**

Early intervention in cerebral palsy could improve motor outcome but is only possible following early identification of those affected. There is a need for training of healthcare professionals (HCPs) in early detection of atypical motor development. We developed a video-based e-learning course - Training in Early Detection for Early Intervention (TEDEI) - to address this need. We evaluated whether participation in the course improved knowledge and changed behaviour of HCPs.

**Methods:**

Participants were 332 HCPs (38% physiotherapists, 35.8% occupational therapists), predominantly UK-based (83.7%). Analysis of training effects used mixed methods and followed Kirkpatrick’s model, first assessing “Reaction” through a feedback questionnaire involving Likert scale and free text responses (*n* = 141). “Learning” was assessed through multiple choice questions (MCQs): all 332 HCPs completed a pre-course quiz of 6 MCQs followed by the course, then a 16 item post-course quiz including the 6 pre-course questions. “Behaviour” was assessed through in-depth qualitative interviewing of 23 participants.

**Results:**

“Reaction”: TEDEI was found to be effective, engaging and well structured. “Learning”: Scores improved significantly between the pre-course and post-course quiz, median improvement 1/6 (z = 5.30, *p* < 0.001). HCPs also reported a perceived improvement in their knowledge, confidence and ability. “Behaviour”: HCPs could see how TEDEI would improve their clinical practice through having an assessment framework, ways of working better with parents, and developing observational skills useful for tele-health assessments.

**Conclusion:**

Our brief e-learning course on early detection for early intervention was viewed positively, improved knowledge and showed potential for positive changes in practice. Kirkpatrick’s model provided a useful framework for undertaking this evaluation.

**Supplementary Information:**

The online version contains supplementary material available at 10.1186/s12909-023-04113-7.

## Background

Motor impairments in children are often attributable to early acquired brain injury, in particular cerebral palsy (CP), which has a prevalence of 2.1/1000 live births [1]. Impairment of motor function often has lifelong adverse effects on activities of daily living, social relationships, quality of life and self-esteem [2].

Major developmental changes occur in the brain and spinal cord during the first 2 years of life [3]. Current consensus supported by an emerging evidence base favours early intervention to improve motor and other developmental outcomes following early acquired brain injury [4]; this is reflected in policy [5]. Early referral and prompt diagnosis are also important to optimise parental support [6].

Early intervention relies on early identification and timely referral of infants with emerging atypical motor signs. Surveillance programs exist for infants born prematurely, who are at high risk of CP [7]. However, 45% of all infants with CP are term-born – they are often not identified as being at risk, and therefore receive routine care [8]. With the appropriate knowledge, skills and access to appropriate cranial imaging, infants with emerging CP can be identified in the first 6 months of life [9]. Despite this, the mean age at referral for diagnosis of CP is 16.6 months [10].

**Fig. 1:**
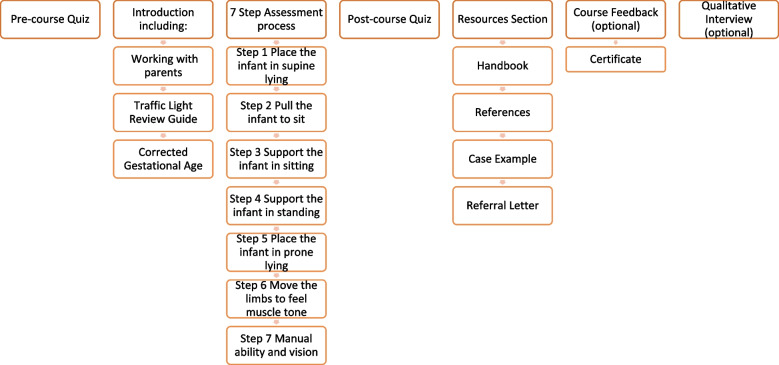
Overview of TEDEI course structure

Motivated by this knowledge and by feedback from parents of infants with CP [11, 12], we developed an e-learning course – Training in Early Detection for Early Intervention (TEDEI) (Fig. 1). The course is tailored towards frontline healthcare professionals (HCPs) and aims to improve awareness of early signs of emerging movement difficulties. It highlights examples of typical and atypical movements and indicates when to refer infants for further investigation.

We aimed to evaluate learning from TEDEI using Kirkpatrick’s four stage model [13], focussing on the first three stages (Reaction, Learning, and Behaviour, here capturing the scope to change behaviour). The final step, Impact, would require evaluation of infant outcomes and was beyond the scope of this project.

## Methods

### Design and setting

The study used a mixed methods design. Ethical Approval was given by Newcastle University Research Ethics Committee (reference 18,219/2019). The course was hosted online by Newcastle University and widely promoted with support from the University Enterprise team. Data was collected between 21.1.2020–30.09.2020.

### Participant selection

Participants were registered HCPs or students. A short paragraph outlining the course features and intended audience was accessed prior to enrolment. Potential participants were informed at the start that if proceeding, their anonymised data including quiz scores would be used for evaluation. After the post-course quiz and feedback, course completers accessed an invitation to take part in a 30-minute semi-structured qualitative interview about the course. If they agreed to being contacted with further information about the qualitative interview, they provided their contact details (phone and/or email) and were subsequently contacted by a member of the research team, provided with an information sheet, and written fully informed consent obtained prior to taking part. We sampled purposively, to include different professional backgrounds and levels of prior experience. We aimed to interview 30 participants, or to continue until data saturation was achieved.

### Procedure

**Fig. 2:**
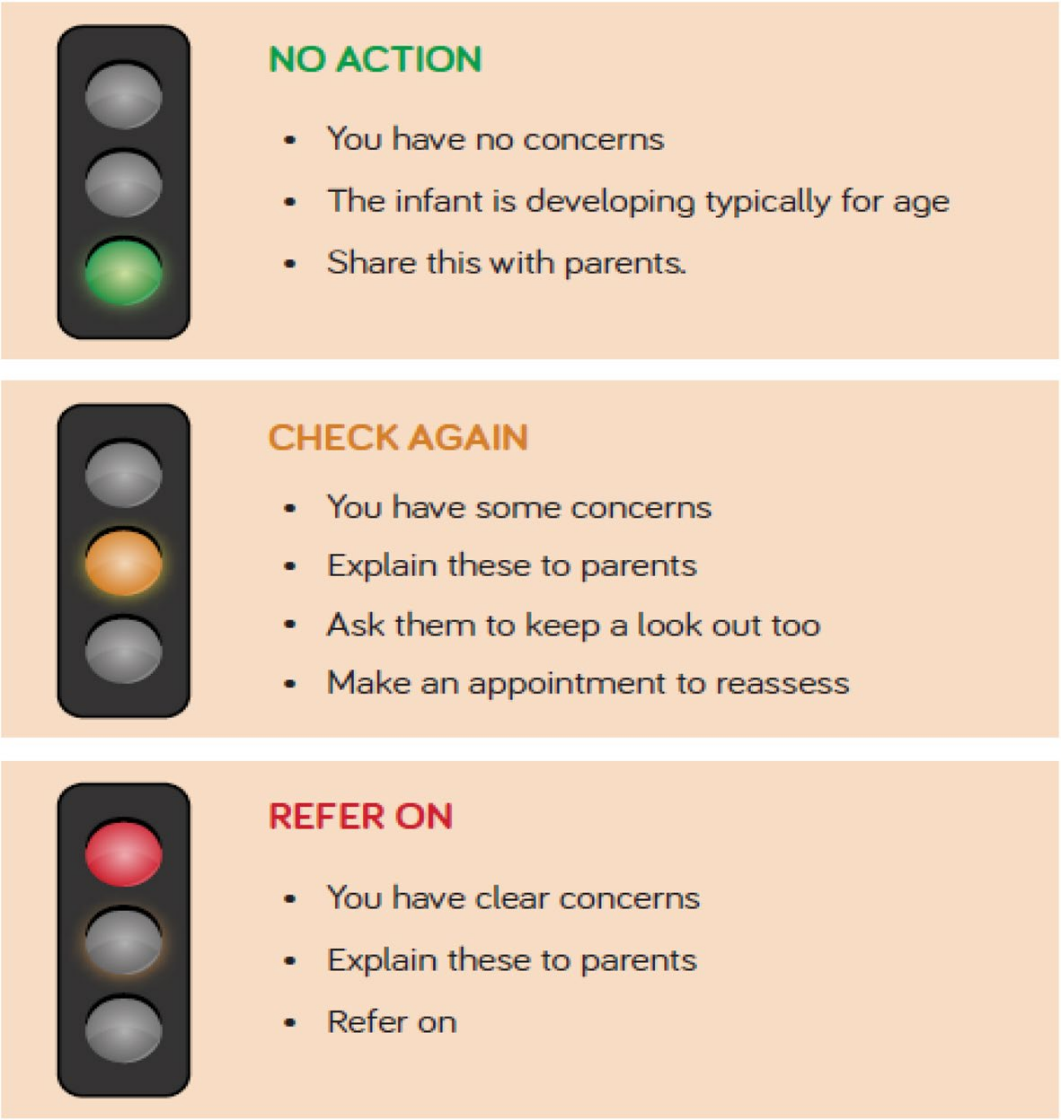
Traffic light system

Participants provided information on the country in which they currently work; profession; and years of experience working with infants. They then completed a six-item pre-course quiz (single attempt) comprising videoed clinical scenarios showing infants with suspected movement difficulties. They were required to select from three options: ‘This infant is moving typically for age’; ‘This infant may not be moving typically for age’ and ‘This infant is moving atypically for age’. No feedback was given at this stage other than their quiz score. The aim was to arouse their curiosity and focus regarding the intended learning [14]. The three-option approach described above was followed through in the course content, using a simple traffic light system (Fig. 2).

Participants undertook the course at their own convenience (Fig. 1; Table 1). They could access and download a course manual to accompany the course: a version of this manual with photographs removed for confidentiality is provided for reference as supplementary material. On course completion, participants sat a 16-item post-course quiz which included the six pre-course questions plus ten other similar questions and followed the same format, though the order of presentation was randomised. Post-course quiz scores were provided alongside explanations and an opportunity to review the videos to promote further learning [15]. Participants could make unlimited attempts at the post-course quiz, allowing development of enactive mastery [16]. They then completed a 10-item feedback questionnaire, with 7 items requiring 5-point Likert-scale responses relating to perceived course quality and effectiveness; an item regarding completion time; and two free text items highlighting the top three learning points and ways in which the course could be improved. A certificate of course completion was then provided.

**Table 1:** Description of TEDEI course

TEDEI is a self-contained module delivered as structured guided asynchronous e-learning through brief online lectures. Following an introduction, the course provides a 7-step structure for infant assessment, which includes review in a variety of positions (e.g. supine; supported sitting), plus muscle tone, manual ability and vision. Following the principles of constructive alignment [15], the course and quiz are rich in video material, in line with the intended learning outcome of increasing recognition of atypical infant movements. Real-life (anonymised) quotes from parents, and infant videos, provide authenticity and contextualisation to prior clinical experiences, facilitating higher order thinking [16]. A simple “Traffic Light System” (Fig. 2) is used to decide for each scenario whether no action, further review or prompt referral is indicated. The course lasts around 2.5 hours and is divided into short sections which can be completed individually. Audio voice-overs and written transcripts facilitate a multimodal learning approach to suit a range of preferred learning styles.	

### Qualitative interviews

Interviews were conducted by MJ (a female final year BSc honours undergraduate student with prior research internship experience and who first undertook a training module in qualitative research) and JP (a female paediatric occupational therapist with many years of research experience including experience of qualitative interviewing, and an MPhil). Interviewees did not have a prior working relationship with those interviewed but introductions were made at the start of the interview process. Participants were made aware of the goals of the research at the start of the interview. In terms of reflexivity, JP had led on the course development but was keenly aware of the need to avoid bias towards a favourable outcome in her questioning and interpretation. MJ had not been involved in development of the course and had no prior detailed knowledge of the research field; she was therefore in a position of equipoise.

Interviews took place either face to face in a quiet office setting or by telephone, at the convenience of the interviewee. They were held on a 1:1 basis, with no observers or other non-participants present. No repeat interviews were undertaken.

Interview content was shaped by a topic guide; this was not formally pilot tested but was reviewed by research team members prior to implementation. Interviews began with discussion about the participants’ professional background and prior knowledge of the subject area. This was followed by consideration of what the participant had gained from the training and how it had influenced, or could influence, their practice. The course structure and content were also discussed. Interviews were audio recorded, transcribed verbatim and pseudonymised prior to analysis. Due to the straightforward nature of the interviews, separate field notes were not undertaken and transcripts/findings were not returned to participants for comment.

### Analysis

Participant data was included where both pre- and post-course quizzes were completed, and no data items were missing. Quantitative data was analysed using IBM SPSS Statistics v26. Data were summarised using descriptive statistics. We used Kirkpatrick’s training evaluation model to structure our analysis. The first level of this evaluation, “Reaction”, assesses to what extent participants found the training engaging and relevant. The feedback questionnaire produced the core data for this section, supplemented by the qualitative interviews. To evaluate level two ("Learning"), we compared pre- and post-course quiz scores for the six repeated scenario questions, using the Wilcoxon signed rank test, as well as comparing the complete pre and post quiz scores. Finally, to evaluate “Behaviour” (reported change in practice or scope for change, as project resources did not extend to capture of observations of behavioural change), we used data from the qualitative interviews.

Qualitative interviews were analysed using a form of thematic analysis known as the Framework approach [17, 18]. The first step was to gain familiarity with the interviews; this was followed by coding (Table S1 shows the coding structure). Independent coding and cross-checking of transcripts was undertaken (EO and JP reviewed all transcripts; MJ reviewed 13 transcripts), and the research team jointly discussed interpretations of key issues emerging from the data. This led to development of a working analytical framework, which was used to index the transcripts and chart the data into the framework matrix, with reference to illustrative quotations. From this matrix (created in Microsoft Excel 365), common themes and sub-themes could be identified and described.

## Results

**Fig. 3:**
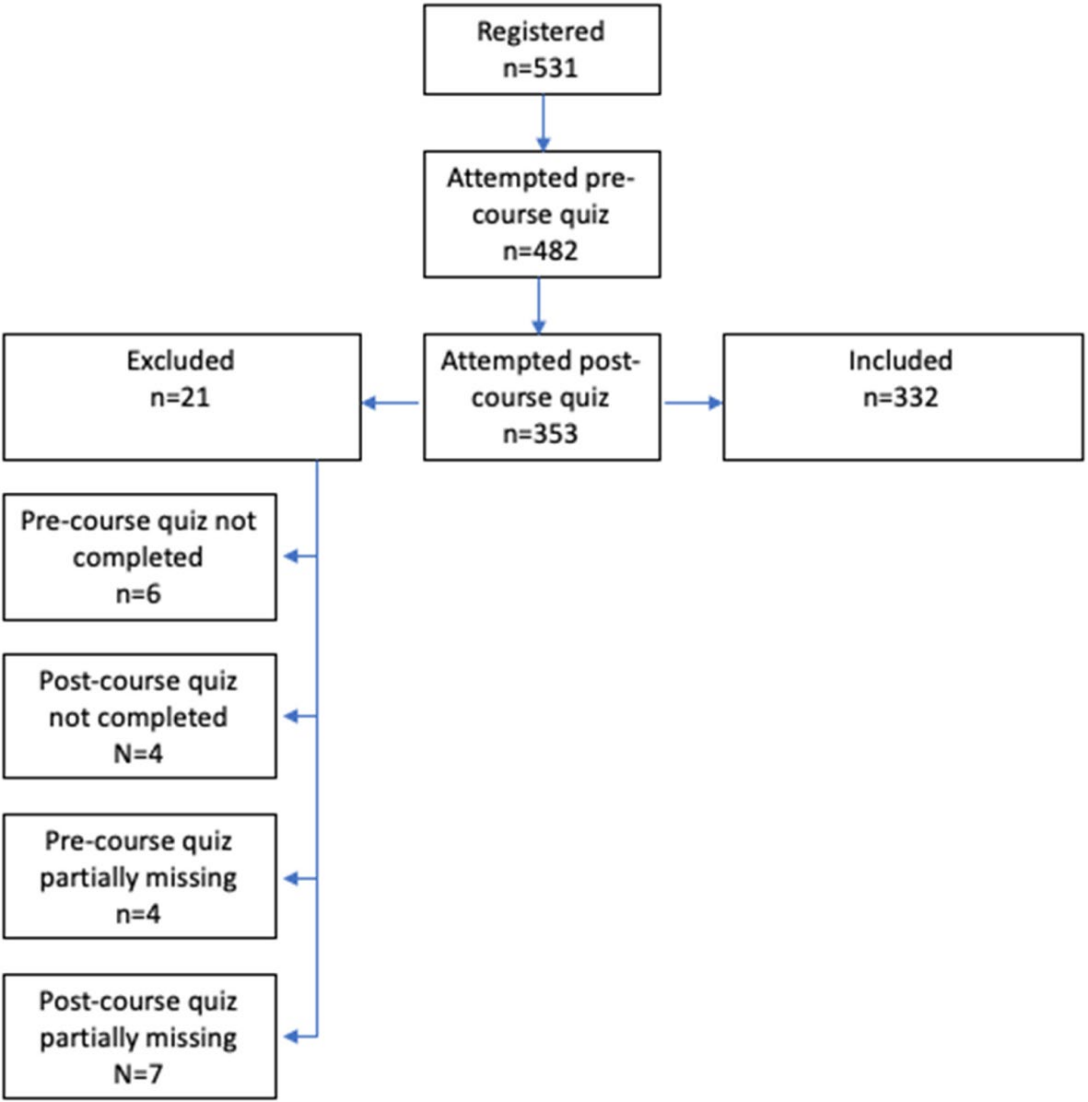
Participant Flowchart

At the time of evaluation, 531 users had registered for the course. Of these, 353 had completed the pre-course quiz, learning module and post-course quiz, of whom 21 were excluded due to missing data (Fig. 3). Thus, pre- and post-course quiz data from 332 participants (“course completers”) was analysed.

258 (77.7%) of course completers were therapists: (126 physiotherapists, 119 occupational therapists, 10 therapy assistants, 3 speech and language therapists). 34 (10.2%) had a nursing/midwifery background, including community nurses, health visitors, nursery nurses, hospital-based nurses, GP practice nursing and others who stated their professional body as the Nursing and Midwifery Council but did not further elaborate. 19 (5.7%) were doctors including Paediatricians, Neonatologists, Specialty Trainees, GPs and medical students. Finally, 21 (6.3%) reported their professional background as “Other”. Course completers self-reported a median of 4 years (IQR = 10) of professional experience working with infants, ranging from less than one to over 30 years. They were practising across 16 different countries, with 83.73% (*n* = 278) based in the UK. 5.12% (*n* = 17) were based in non-native English-speaking countries.

The post-course feedback questionnaire was completed by 141 participants (“questionnaire respondents”), i.e., by 42.5% of course completers. Professional backgrounds were comparable: 77% of this group were also therapists, with a similar distribution of prior experience.

All 27 participants who agreed to take part in a qualitative interview were approached for consent. Four of these did not respond to this approach. Qualitative interviews were undertaken by 23 participants (“interviewees”), of whom 7 were physiotherapists, 4 occupational therapists, 6 doctors, 2 medical students, 1 midwife, 1 health visitor and 2 research students. Nine interviews were undertaken face to face, and the rest by telephone.

Thirteen interviewees considered themselves to have a high level of prior knowledge and experience of infant movement difficulties.

### Level 1: “reaction”

**Fig. 4:**
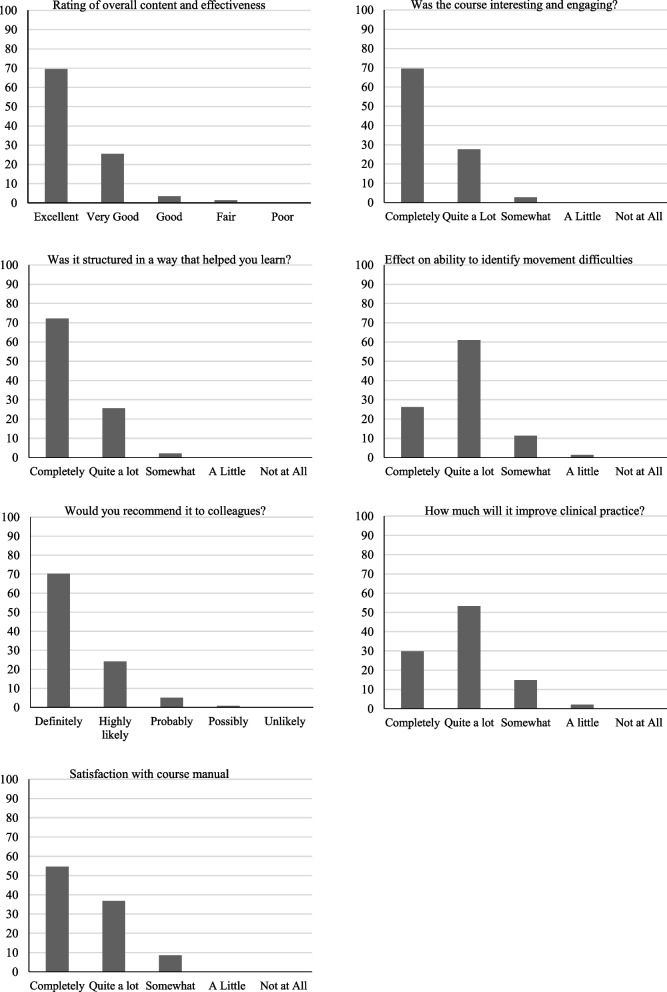
Participant questionnaire feedback

Figure 4 summarises findings from Likert-scale items in the feedback questionnaire.

Interviewees reported the course to be valuable and professional, with relevant, specific and comprehensive content. TEDEI was considered to provide a good understanding of the topic and was viewed as achieving its aim to facilitate early detection. Several interviewees thought TEDEI filled a gap in currently available learning resources. They commented that there were few resources teaching HCPs to assess movement in infants that included visual examples of typical and atypical movements, direct explanations and that were easily accessible: most courses are in-depth, delivered face-to-face, costly and require travel.

"You don’t have much [in the way of] resources that you can access easily around this area. I want to stress that point”.

Paediatric Physiotherapist, 2 years’ experience

Interviewees found the course to be engaging and enjoyable:

“I did not feel like I wanted to just zoom to the quiz to get my certificate. I felt like I really wanted to take my time and go through each section”.

Paediatric Occupational Therapist.

Interviewees found the content concise and clear, the structure logical and simple and the traffic light system helpful. Overall, interviewees found the multiple media types (video, narration, text and transcripts) useful to facilitate learning, and that there was a good balance between these. One interviewee was sometimes distracted by having materials presented in multiple formats simultaneously:

“Sometimes, the video was running while somebody was speaking and then it was like, “Oh, I’ve missed the video because I was listening, and not looking within those sections”

Paediatric Physiotherapist

The video examples were regarded by many interviewees and questionnaire respondents as the most important part of the course. It was particularly useful to see the difference between typical and atypical movements, and to be able to review the materials multiple times. The video examples were described as having varied and relevant content and were an engaging learning resource, allowing HCPs to compare their own technique to that shown. The accompanying downloadable handbook was considered a visually appealing and convenient resource that HCPs could refer to when assessing infants. Interviewees liked the summary of the course content to which they could add their own notes. The course was thought to be beneficial to a range of HCPs, particularly for trainees and less experienced practitioners, to form a basis for developing skills and confidence; and to provide a framework for those who may have difficulty gaining hands-on experience, whilst also being good value for money.

Course completion time was 2–4 hours in 83 (58.9%) of questionnaire respondents, with 18 completers taking less time than this, 23 taking longer and 17 not specifying. Most interviewees found the course length appropriate though some felt that it was too long for busy HCPs with diverse caseloads. The ability to “dip in and out” of the course mitigated against this. It was also considered valuable to be able to revisit the material even after completion.

Some participants suggested ways in which the course could be expanded in future. These included covering a wider age group, including even more videos, and covering other aspects of development.

### Level 2: “learning”

All questionnaire respondents felt that the course had improved their ability to identify movement difficulties in infants (Fig. 4). Percentage results between pre-course (6 items) and post-course tests (all 16 items; first attempt) improved for 286 (86.1%) of course completers. Mean scores for the pre-course and post-course quizzes were 51.5 and 74.6% (first attempt) respectively, with a mean difference of 23.1% (Z = − 14.066, *p* < 0.001). Fifty participants undertook the post-course quiz a second time (ten of whom made a third and one a fourth attempt). 38/50 improved their post-course quiz scores on the second compared with the first attempt.

A Wilcoxon signed-rank test was conducted to determine the effect of the course on performance in the 6 repeated MCQs. Results showed a median improvement of 1 correct response between the pre-course quiz (median = 3/6) and the post-course quiz (median = 4/6), z = 5.30, *p* < 0.001.

Interviewees found the quizzes useful for consolidating, testing and gauging improvement in knowledge, and one suggested that the pre-course quiz helped identify areas to focus on within the course. One interviewee found it difficult to make decisions about infants based on short video snapshots.

Interviews identified knowledge, structured assessment and reassurance as the main learning points. Knowledge about the ages at which specific movements emerge was improved. The course included new information and perspectives even for some experienced HCPs. Teaching on how and why to correct for gestational age was appreciated.

Feedback about the structured, seven step assessment process was overwhelmingly positive. Interviewees found the steps to be straightforward, logical and well-structured.

Some experienced interviewees commented that while the course did not directly improve their knowledge, it provided reassurance regarding their existing knowledge and assessment techniques.

### Level 3: “behaviour”/reports of change in practice and scope for change

All questionnaire respondents indicated that their clinical practice would be improved by the course (Fig. 4). There were also reports of actual change in practice. One interviewee now prepares for appointments by reviewing the course content and uses the handbook as a prompt; another experienced interviewee felt empowered to make greater use of her observational skills. Some interviewees had already related the material to current cases and applied their new knowledge to practice:

“The family that I was talking to, the baby had had a neurological impact at term, and everything the parents were saying about her not using her left arm as much, it just rang all those alarm bells from the course videos”

Experienced physiotherapist

Others considered using the course to facilitate their role in training colleagues:

“It’s certainly made my job easier because … ..I obviously do all the training and I don’t have a package for it”

Experienced physiotherapist working on neonatal unit

Participants could see how the course could lead to earlier referral of infants with emerging movement difficulties, i.e., the content had face validity.

#### Working with parents

Interviewees voiced that the course caused them to change the ways in which they communicate with parents, listening more to their concerns and observations, and altering their language to encourage them to talk.

“I think a lot of parents are just not listened to … ..and parents often don’t feel confident saying ‘oh well I’ve noticed this’ when actually that’s really really key … when I’ve been speaking to parents instead of just saying ‘this is what we think’ I’ve said ‘well, what do you think, what have you noticed, are you concerned?’ so I think even just changing the language I’m using, has been really, has happened as a result of this.”

Experienced Occupational therapist

#### Confidence

TEDEI training was seen to increase confidence, as a mediator for behaviour change. Several interviewees felt more confident about referring infants for further assessment; they could use the course materials to give credence to their concerns, and model the language used in communicating concerns to parents and other professionals.

"I wouldn’t have been really confident before at all but now I would feel - I probably would take somebody with me the first few times but I would feel that I could lead on it and work through it in front of somebody, in front of parents and look confident enough to do that."

Occupational therapist relatively new to the field

#### Training for tele-health assessment

The COVID-19 pandemic led to rapid changes in practice within healthcare, including a vast increase in remote consultations. Nine of the 23 interviews took place after the start of the pandemic. Interviewees commented that the course’s focus on observation was extremely valuable for tele-health assessments.

“The fact that I was restricted to tele-health, it really made me look at movements even more.”

Experienced paediatric physiotherapist

## Discussion

The TEDEI course improved recognition of early signs of atypical motor development in infants by HCPs. The course helped learners from a range of clinical backgrounds and levels of experience to improve their understanding of which presentations required immediate referral, which required review, and which were within the limits of typical development, as well as providing a framework for assessment, and highlighting good practice in communication with parents and other HCPs. Kirkpatrick’s model provided a useful framework for evaluation.

Other e-learning resources for HCPs have also yielded positive feedback, potential improvements in knowledge base and changes in practice [19]. A recent systematic review concluded that e-learning was at least as effective as traditional learning approaches in improving HCP behaviour [20]. A major advantage of e-learning over face-to-face teaching is reach. At the time of writing, our course has been accessed by over 800 participants based in the UK and abroad. The course was accessible during the COVID-19 pandemic: the need to undertake remote, video-based assessment made the course timely, though we acknowledge that face to face clinical experience cannot be fully replaced by e-learning [21]. The video-rich content was noted as a strength; the broader literature also indicates that students find videos a helpful component of clinical skills training [22]. Videos can accelerate learning by greatly increasing the rate of exposure to clinical signs.

Whilst our analysis focusses on evaluation of learning, factors other than course content influence the quality of an e-learning product. These include the design of graphical and visual information; “interaction usability” (ease of use including navigation) and accessibility for learners with differing needs [23]. Positive feedback was also obtained regarding these aspects: we benefitted from the input of an e-learning technologist and from undertaking multiple pre-launch test runs.

Some limitations should be noted. Firstly, we do not have feedback from those who did not complete the course; furthermore, fewer than 50% of course completers provided questionnaire feedback. The breakdown of respondents in terms of predominant professional background and range of experience indicated that questionnaire completers were representative of the broader group in these respects, though it is possible that those providing feedback had particularly positive experiences. Secondly, not all respondents provided adequate detail regarding their professional background. Thirdly, as the course was self-paced, some learning may have been achieved between online sessions. For example, participants may have become aware of their knowledge gaps due to commencing the course; they may then have addressed some of these prior to undertaking the post course quiz. Participants may also have sourced some of the additional resources recommended and learned from these. Both scenarios align with the overall aim of the course and our hope would be that such learning would continue even after course completion.

Our long-term goal is that all infants with emerging movement disorders are identified and referred promptly for further support including effective early intervention. We acknowledge the enormity of this task, which also requires secondary care professionals to have advanced diagnostic skills, and an expansion of the early intervention evidence base.

Upskilling front-line health care professionals in early identification of potentially affected infants remains a critical step towards this goal. We recognise that a two-hour course can only provide an accessible introduction to early detection for early intervention, and hopefully support clinical reasoning. However, we are aware that many frontline healthcare practitioners have only very limited time and funds to direct to upskilling on this single aspect of healthcare. We signpost within the “Resources” section of the online course to more detailed information including books and courses on child development and infant movement assessment. Our hope is that a short introductory course can realistically be accessed by many practitioners and thus make a positive difference to early detection and referral rates, ideally also promoting ongoing learning and improvement.

In conclusion we have shown how a simple e-learning package can improve knowledge, confidence and change reported behaviour and behavioural intent regarding early detection of movement difficulties in infants.

## Supplementary Information

**Additional file 1.** Course manual (photographs removed).

**Additional file 2: Table S1.** Coding structure.

## Data Availability

The datasets used and/or analysed during the current study are available from the corresponding author on reasonable request.

## Abbreviations

TEDEI: Training in Early Detection for Early Intervention
HCPs: Healthcare Professionals
MCQs: Multiple Choice Questions
CP: Cerebral Palsy
