# Supplementary material for: Evaluation of the Training in Early Detection for Early Intervention (TEDEI) e-learning course using Kirkpatrick’s method
Source: BMC Med Educ. 2023 Feb 27;23:129. doi: 10.1186/s12909-023-04113-7 (PMC9968638; doi:10.1186/s12909-023-04113-7)
Supplement: Supplementary file 1 — Additional file 1. Course manual (photographs removed). [file 12909_2023_4113_MOESM1_ESM.pdf]

Training in Early Detection for Early Intervention

An e-Learning Course for Healthcare Professionals Working with Infants

COURSE HANDBOOK

FUNDED BY
NIHR | National Institute for Health Research

© 2019 Newcastle University

Training in Early Detection for Early Intervention

CONTENTS

Introduction 5
What is TEDEI? 5
Who has TEDEI been developed for and why? 5
How to access the online course 5
Background to TEDEI 6
TEDEI Online Course Structure 7
Listen and talk to Parents 8
What to do if you see/feel/hear about something of concern 8
TEDEI Traffic Light Actions 9
Corrected Gestational Age (CGA) 10
TEDEI's 7 steps to observe and feel posture, movement and tone 12
Step 1 Place the infant in supine lying 14
Step 2 Pull the infant to sit 16
Step 3 Support the infant in sitting 17
Step 4 Support the infant in standing 18
Step 5 Place the infant in prone lying 19
Step 6 Move the limbs to feel muscle tone 20
Step 7 Observe manual ability and vision 22
TEDEI Reading List 24
Case example of how to explain to parents that you have concerns 26
Case example of how to write a referral letter 27
Index of Video Clips 28
Acknowledgements 29

© 2019 Newcastle University

© 2019 Newcastle University

INTRODUCTION

This booklet is designed to be a companion document to the TEDEI e-learning course to use as you wish. It contains space for you to make notes and may be a useful reference document once you have completed the training. The video examples are referenced allowing you to locate them to look at them again. You may wish to refer to them if you have concerns about an infant you have seen.

What is TEDEI?

TEDEI (Training in Early Detection for Early Intervention) is an e-learning course offering training on typical motor development in term and preterm infants, early warning signs of high-risk infants and emerging signs of motor difficulties. It focusses in detail on neurological presentations. We have made this training package primarily to help healthcare professionals develop skills in the early detection of atypical motor development in infants.

TEDEI includes a simple "traffic light" guide on what action to take when motor difficulties are observed with examples of how to approach identified difficulties with families.

A certificate of course completion will be provided for the user's own professional development records.

Who has TEDEI been developed for and why?

TEDEI has been designed principally for frontline community healthcare professionals (HCPs) including health visitors and general practitioners, practice nurses and nursery nurses but is relevant to many others including nurses, occupational therapists, paediatricians, physiotherapists, play specialists, and Portage workers.

TEDEI provides a detailed introduction to detecting atypical motor skill development in infants from term age up to six months old. By *term age* we mean due date, give or take a couple of weeks; so for preterm infants this won't be the same as their date of birth. So for preterm infants you must use their corrected age (see page 10).

Developing skill in the early detection of atypical motor development requires a good understanding of typical motor development and we recommend that participants have this prerequisite knowledge before commencing this course. TEDEI does not cover other aspects of child development such as communication and social interaction. It is important to consider these and many other factors when seeing an infant.

More in depth courses are available from other providers and some information can be found in the 'Further Information' section at the end of this booklet. You should use this TEDEI training to support your clinical reasoning.

How can Healthcare Professionals (HCPs) access the course?

Our long-term aim is that as many infants as possible are identified early, and entered into effective early intervention programs. TEDEI is hosted on Newcastle University's Short Online Courses Platform , and the e-learning team have provided expert input to the course development:

http://cpd.ncl.ac.uk

This platform is available to registrants worldwide.

 © 2019 Newcastle University

Training in Early Detection for Early Intervention

An e-Learning Course for Healthcare Professionals Working with Infants

Designed at Newcastle University by:
Janice Pearse with support from Dr Anna Basu

Produced by:
Ashley Reynolds

About us:

Janice Pearse is an Occupational Therapist with many years of research and clinical experience in the management of children with cerebral palsy and has presented and published research in hemiplegia. She co-developed the Early Therapy in Perinatal Stroke (eTIPS) therapy approach[9] and supported parents in its use within the eTIPS pilot feasibility study[10].

Dr Anna Basu is a Paediatric Neurologist with extensive research experience in neurological disorders of children, particularly those of cerebral palsy. Her research focusses on assessing and intervening to improve upper limb function in children with, or at risk of developing, hemiplegic cerebral palsy. She was the Chief Investigator (CI) of the Early Therapy in Perinatal Stroke (eTIPS) pilot feasibility mixed methods study[10]. Dr Basu is the Neurology advisor to the UK-based EI SMART multidisciplinary group, rethinking our approaches to early intervention for high-risk infants in the UK.

Ashley Reynolds is an e-Learning Technologist with over 18 years' experience in the design and production of e-Learning, Mr Reynolds has produced hundreds of CPD courses for organisations including several pharmaceutical manufacturers and large retailers.

Acknowledgements:

We would like to thank all the families who consented to video clips of their infants being used to create this training resource. We would also like to thank: Jessica Baggaley, Patricia Dulson, Grace Edmonds, Ceit Jesmont, Claire Marcroft, Veronica O'Malley and Helen Rowland for reviewing the material; and Louis Francis for assisting with editing the video clips.

Funded by:

© 2019 Newcastle University

TEDEI Online Course Contents and Approximate Times to Complete each section

Section	Step/Contents	Approximate Time to complete
Introduction	Including background, what to do if you see something of concern, TEDEI's Traffic Light Actions, and Corrected Gestational Age (CGA)	
Step 1	Place the infant in supine lying	
Step 2	Pull the infant to sit	
Step 3	Support the infant in sitting	
Step 4	Support the infant in standing	
Step 5	Place the infant in prone lying	
Step 6	Move the limbs to feel muscle tone	
Step 7	Observe manual ability and vision	
Quiz	Test Yourself	
Resources	Here you will find references, and other useful information.	

© 2019 Newcastle University

Background to TEDEI:

Motor impairments in childhood are often attributable to injury to the developing brain around the time of birth. Cerebral Palsy (CP) is the most common of these, with many subtypes and a prevalence of 2.1 per 1000 live births [1]. The effects are variable but impairment of motor function is common to all. The effects can be seen throughout the lifespan and often impact upon mobility, activities of daily living, social relationships, quality of life and self-esteem. Other conditions which can affect infants may also lead to motor impairments including genetic syndromes and muscle disorders.

Major developmental changes occur in the brain and spinal cord during the first 2 years of life [2], with most gross motor potential achieved within this period. Children with CP reach 90% of their gross motor potential by age 5 years [3] and consequently the consensus of current opinion is that intervention for children with CP should start as early as possible [4,5]. Early intervention during this period has the potential to improve motor outcome to a greater extent than intervention occurring once these developmental changes are largely over and therefore is critical to the well-being of infants and their families[6].

Early intervention relies on early identification and timely referral of infants with emerging signs of atypical development. It is well known that infants born prematurely are at risk of developing CP with the risk increasing with earlier gestational age [4]. 45% of all children born at term and later diagnosed with CP (usually around age 5 years) were not identified as being at "high risk" of CP as infants[6]. Parents often suspect that their infant's development is not as it should be and want to know if there is a reason for this. In one study 86% of parents whose child went on to receive a diagnosis of CP thought that their infant was not developing as they should be, yet a health professional noticed this first in only 16% of cases[7].

TEDEI was developed at Newcastle by Mrs Janice Pearse with support from Dr Anna Basu, in response to a need for improved quality of early detection and early intervention for infants with brain injuries, which was identified during Dr Basu's NIHR-funded Career Development Fellowship. The problems around early detection and early intervention were highlighted again and again by parents of affected infants in our focus groups and workshops, parents of participants in our intervention study, and also through our national survey, using perinatal stroke as an exemplar [8-10]. The educational materials we have developed are rich in video content, provided with parental consent. The use of video is a very effective way of demonstrating what to look out for, and is a powerful but simple teaching tool to effect change. Achieving improved detection of emerging motor difficulties in young infants is critical to supporting families and providing affected infants with timely intervention. Early provision of support is also critical to the well-being of infants and their families.

In April 2019 a consensus paper was published entitled "International expert recommendations of clinical features to prompt referral for diagnostic assessment of cerebral palsy"[11]. The authors sought agreement from international experts on which clinical features detect cerebral palsy and the clinical 'warning signs' to monitor over time. It also makes referral recommendations for primary health care professionals to know when to refer on to specialised health care services. TEDEI broadly follows these recommendations, though we use some specific examples where referral to specialist services is appropriate sooner than the general time frames recommended in the paper.

References

1. Himmelmann K, Uvebrant P. **The panorama of cerebral palsy in Sweden. XI. Changing patterns in the birth-year period 2003–2006.** *Acta Paediatrica.* 2014;103(6):618-624.

2. Eyre JA, Smith M, Dabydeen L, et al. **Is hemiplegic cerebral palsy equivalent to amblyopia of the corticospinal system.** *Ann Neurol.* 2007;62.

 © 2019 Newcastle University

3. Morgan C, Darrah J, Gordon AM, et al. **Effectiveness of motor interventions in infants with cerebral palsy: a systematic review.** *Dev Med Child Neurol.* 2016;58(9):900-909.

4. Spittle A, Orton J, Anderson PJ, Boyd R, Doyle LW. **Early developmental intervention programmes provided post hospital discharge to prevent motor and cognitive impairment in preterm infants.** *Cochrane Database of Systematic Reviews.* 2015(11).

5. Novak et al. **Early, Accurate Diagnosis & Early Intervention in Cerebral Palsy: Advances in Diagnosis and Treatment.** *JAMA Pediatr.* 2017;171(9):897–907.

6. McIntyre S, Morgan C, Walker K, Novak I. **Cerebral Palsy - Don't Delay.** ***Developmental Disabilities Research Reviews.*** 2011;17(2):114-129.

7. Baird G, McConachie H, Scrutton D. **Parents' perceptions of disclosure of the diagnosis of cerebral palsy.** *Archives of Disease in Childhood.* 2000;83(6):475-480.

8. Basu AP, Pearse JE, Baggaley J, Watson RM, Rapley T. **Participatory design in the development of an early therapy intervention for perinatal stroke.** *BMC Pediatr.* 2017;17(1):33.

9. Basu AP, Pearse J, Watson R, et al. **Feasibility trial of an early therapy in perinatal stroke (eTIPS).** *BMC Neurol.* 2018;18(1):102.

10. Marcroft C, Tstutsumi A, Pearse JE, Dulson P, Embleton ND, AP. B. **Current therapeutic management of perinatal stroke with a focus on the upper limb: a cross sectional survey of UK physiotherapists and occupational therapists.** *Phys Occup Ther Pediatr* 2018.

11. Boychuck, Z. , Andersen, J. , Bussières, A. , Fehlings, D. , Kirton, A. , Li, P. , Oskoui, M. , Rodriguez, C. , Shevell, M. , Snider, L. , Majnemer, A. & GROUP, T. P, **International expert recommendations of clinical features to prompt referral for diagnostic assessment of cerebral palsy.** Dev Med Child Neurol. 2019

© 2019 Newcastle University

At the Appointment:

Listen to Parents

1. Listen Carefully
2. Guard against dismissing concerns
3. Discuss any issues of concern in a realistic, but sensitive manner.
4. Tell parents they aren't to blame.
5. Say what action you will take.

TALK to Parents

1. Ask: "Do you have any worries about how your baby is
   - moving?
   - seeing?
   - hearing?
   - interacting with you?"
2. If parents describe any concerns you should watch out for these. Ask them to show you any relevant video they may have taken of their infant
3. Explain that you will both watch and feel how the infant moves.

> Be vigilant for other signs if you hear about, feel, or see something of concern.
>
> If you identify more than one issue of concern then you should consider referring the infant on.

What to do if you see or feel something of concern:

See / feel it once - go back and CHECK AGAIN

- Talk to parents if you see something of concern. You should explain it sensitively and clearly. Page 26 has an example of how you might explain your observations to parents
- Tell parents you expected to see it.
- Ask parents if they have noticed it too.
- Suggest that parents watch out for it in case it happens again. They should video it if possible and keep a record of when it happens.
- Be vigilant for other signs of delay.
- If you identify an issue you should **always** arrange another appointment to reassess.
- You may have significant concerns that justify referring on at this stage
- If in doubt discuss your concerns with a colleague

See / feel it twice - REFER ON

- You should ensure the infant is referred on as soon as possible to an appropriate service or professional to ensure that the infant receives timely intervention.
  Page 27 has an example referral letter.

© 2019 Newcastle University

Traffic Light Actions

To help you with decision making we have summarised the actions you should take into a traffic light system. These actions are used with the video clip examples within TEDEI:

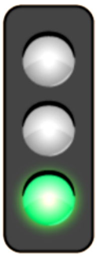

GREEN

NO ACTION

- You have no concerns
- The infant is developing typically for age
- Share this with parents.

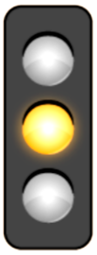

AMBER

CHECK AGAIN

- You have some concerns
- Explain these to parents
- Ask them to keep a look out too
- Make an appointment to reassess

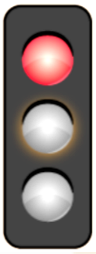

RED

REFER ON

- You have clear concerns
- Explain these to parents
- Refer on

REMEMBER:

If you identify several AMBER issues at one appointment you must consider if this is enough evidence to refer on at this stage.

ALWAYS consider your observations alongside any information parents provide.

IF IN DOUBT discuss your concerns with a colleague.

Who should I refer on to?

TEDEI has been designed principally for frontline community healthcare professionals including HVs, GPS, practice nurses and nursery nurses. If you fall into one of these groups, and identify an infant with motor difficulties, you should refer on to one or more of the following professionals: **paediatrician, physiotherapist, occupational therapist**. Services can vary by area so you may need to find out who is most appropriate to refer on to and what the referral process is. Page 27 has an example referral letter.

Obviously if the infant you are concerned about is already under specialist follow up you may not need to refer on, though it may be advisable to share your concerns with that service to ensure nothing is missed.

Notes:

© 2019 Newcastle University

Corrected Gestational Age (CGA)

A **term** pregnancy is around 40 weeks, +/- 2 weeks; therefore most infants are born between 38 and 42 weeks. Infants born at 37 weeks or less are considered to be **premature**. Premature infants are more likely to reach their milestones later than if they had been born at term. To help account for this, their developmental milestones are measured using their **corrected gestational age (CGA)** instead of their **chronological age**. This measure is used for premature infants to ensure that their development is assessed fairly. Their age is corrected only up until they reach 2 years of age from their **actual date of birth**.

How to calculate Corrected Gestational Age (CGA):

1. Find out the infant's **gestational age** in weeks. This is the total number of weeks from conception up until the infant's birth.
2. Find out what the infant's chronological age is at present. **Chronological age** is the length of time since the **actual date of birth**.
3. Subtract the **gestational age** in weeks from 40 weeks (the length of a term pregnancy). This is how many weeks early that the infant was born.
4. Subtract your answer in step 3 from the infant's **chronological age** worked out in step 2.

The answer from step 4 gives you the infant's **corrected gestational age (CGA).**

CGA Glossary

Actual Date of Birth—the date the infant was born.

Chronological Age is the length of time since the actual date of birth (days/weeks/months/years).

Gestational Age is the total number of weeks from conception up until the infant's birth.

Corrected Gestational Age (CGA) adjusts the age for premature infants.

Term Infants are born at 40 weeks gestation (+/- 2 weeks)

Premature or Preterm Infants are born at 37 weeks gestation or less

Expected Date of Delivery (EDD) is 40 weeks after conception.

© 2019 Newcastle University

Examples of how to calculate Corrected Gestational Age (CGA)

EXAMPLE 1:

1. Charlie was born at **28 weeks** (his gestational age).
2. Charlie's **chronological age** today is **16 weeks.**
3. **40 weeks - 28 weeks = 12 weeks**. Therefore Charlie was born **12** weeks early.
4. **16 weeks - 12 weeks = 4 weeks.**

Charlie's **corrected gestational age** today is **4 weeks.** Therefore Charlie should not be expected to be doing anything more than a **1 month** old infant born at term.

EXAMPLE 2:

1. Amy was at born 34 weeks (her gestational age).
2. Amy's **chronological age** today is **18 weeks.**
3. **40 weeks - 34 weeks = 6 weeks**. Therefore Amy was born **6** weeks early.
4. **18 weeks - 6 weeks = 12 weeks.**

Amy's **corrected gestational age** today is **12 weeks.** Therefore Amy should not be expected to be doing anything more than a **3 month** old infant born at term.

Notes:

© 2019 Newcastle University

Training in Early Detection for Early Intervention

TEDEI's 7 Steps to observe and feel posture, movement and tone.

The more you handle infants, the more aware you will become of how typically developing infants feel and move, which will help you recognise when an infant may be exhibiting movement difficulties. We therefore recommend that you try to do these seven steps with lots of infants. We would suggest the TEDEI sequence as a logical method, but you can do the steps in any order.

- Before you begin you will need space to carry out the 7 steps, a padded mat on the floor is ideal.
- Check that the area is safe and comfortable for the infant, and without distractions. If you are in the family home you may need to ask parents to turn off the TV or move pets out of the room.
- Check that the infant is free to move. Ask the parent to remove the infant's outer clothes to allow you to clearly see arm and leg movement.

STEP 1

Place the infant in supine lying

Lie the infant on his or her back (supine) for a few minutes and observe posture and movement.

STEP 2

Pull the infant to sit

Now gently pull the infant up to sit, observing posture and movement and feeling muscle tone as you do so.

If the infant's head is lagging significantly you must support it with your hands.

© 2019 Newcastle University

Support the infant in sitting

STEP 3 From the pull up into sitting you should carefully reposition your hands to **s**upport the infant in sitting. Feel and observe posture and movement.
Keep hold of the infant throughout this step.

Support the infant in standing

STEP 4 From sitting now lift the infant to stand, feel the muscle tone and observe posture and movement.
Keep hold of the infant throughout this step.

Place the infant in prone lying

STEP 5 Gently lower the infant from standing on to his or her front (prone).
As you do this feel the muscle tone and once positioned on the mat observe posture and movement.

Move the limbs to feel muscle tone

STEP 6 Turn the infant over to lie on his or her back.
Now gently move the limbs to feel the muscle tone.

Observe manual ability and vision

STEP 7 Sit the infant in a baby chair (if available) or ask the parent to support the infant in a sitting position on their lap.
These positions let you look at vision, and from 3 months of age onwards, how the infant uses his/her hands in play (manual ability).

Each step is explained in detail on the following pages.

© 2019 Newcastle University

STEP 1 OBSERVE INFANT LYING ON BACK (SUPINE)

1. Lie the infant on his/her back (supine) on a mat on the floor.
2. Position yourself near the infant's feet to allow you to compare the movements on each side of the body.
3. The infant should be contented and alert . Crying influences movement, and can make it difficult to see usual patterns of movement. Sleepy babies will not move very much.
4. Remove the infant's dummy or pacifier. Gross motor movements are reduced when an infant is sucking.
5. Watch for a while without interacting.
6. In very young infants, typical movements should be *varied* and reasonably *fluent.* Movements on each side of the body should be similar in frequency and fluency.
7. Gently turn the infant's head into the midline to remove the effect of the ATNR .

ASYMMETRIC TONIC NECK REFLEX (ATNR)

ATNR is a primitive reflex found in new-born infants. In typical infant development it disappears by around 5 months of age. Characteristically it is seen when an infant is lying supine with the head turned to one side. The arm and leg on the same side extend, and the arm and leg on the opposite side flex. This position resembles that of a fencer and is sometimes referred to as the "fencing reflex".

It is a **cause for concern** if an infant's movements are dominated by the ATNR beyond 5 months of age. It is important to remember that this is an association, and not a diagnostic finding.

Ref: Hamer, E. G. and Hadders-Algra, M. (2016), Prognostic significance of neurological signs in high-risk infants – a systematic review. Dev Med Child Neurol, 58: 53-60. doi:10.1111/dmcn.13051

Plagiocephaly is where one side of the head is flattened causing it to look asymmetrical. Plagiocephaly is associated with a preference for the head to be turned to one side rather than the other. If a young infant's head is turned to one side it can bring out the ATNR posture. When looking for asymmetry move the infant's head gently into the midline to remove the effect of the ATNR. These illustrations show right-sided plagiocephaly as viewed from top of head.

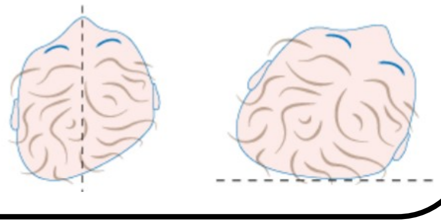

© 2019 Newcastle University

Atypical movements to be aware of when observing infants who are lying supine:

1. **Lack of movement** - you don't see much movement when the infant is awake. Or there is noticeably more (or less) movement in the arms compared to the legs.

2. **Lack of variety of movement** - you see a lot of the same movements repeatedly

3. **Stiff or cramped movements** – both arms and/or legs stiffen at the same time

4. **Jittery or shaky movements** - you see small, involuntary movements that can look like a tremor.

5. **Jerky movements** – you see larger, fast movements which lack fluency.

6. **Asymmetry** – you see clear differences in movement on opposite sides of the body that cannot be attributed to the effect of ATNR or plagiocephaly.

What to do if you see any of these six movement types:

1. Follow the traffic light guidelines on page 9.

2. Be vigilant for other signs of motor difficulties during the remainingTEDEI steps.

In addition, when observing infants who are lying supine, check you are seeing the following developmental milestones:

From 3 months:

- Antigravity movements of limbs
- Visually attentive
- Trunk rotation emerging

From 4 months:

- Hands come together in midline
- Hands are open a lot of the time (not fisted)

From 6 months:

- Hands reach to touch knees
- Trunk rotation marked - rolls on to sides

If an infant is not able to roll on to either side by 6 months, it would be appropriate to refer on.

Notes:

© 2019 Newcastle University

STEP 2 PULL TO SIT

Now gently pull the infant up to sit, observing posture and movement as you do so.

If the infant's head is lagging significantly, you must support it with your hands.

This photograph shows a typically developing 2 month old infant. Note the slight head lag and extended/straight elbows, this is typical for 2 months of age.

What you should expect to see:

- From three months onwards any head lag should be minimal and seen just at the start of the pull-to-sit manoeuvre. Most infants achieve this manoeuvre with their elbows flexed and without any head lag and between 3 and 5 months of age. Flexed elbows mean the infant is helping to pull up into sitting. If elbows are extended, the pull to sit manoeuvre is passive, in other words the examiner is doing all the work.
- By 6 months most infants achieve the pull to sit manoeuvre with elbows, hips and knees bent (flexed), and with a chin tuck. Unless you are seeing all of these things in a 6 month old infant you should check again.

The following are a cause for concern if observed during this pull to sit manoeuvre:

- Head lag beyond 3 months of age
- Extended (straight) elbows beyond 3 months of age

If you see any of these, be vigilant for signs of motor difficulties during the other TEDEI steps.

CHECK AGAIN if the infant demonstrates head lag beyond 3 months of age and/or extended (straight) elbows during this manoeuvre

REFER ON if the infant demonstrates persistent head lag beyond 4 months of age

Notes:

© 2019 Newcastle University

STEP 3 SUPPORT IN SITTING

Support the infant in sitting and observe posture and movement.

What you should expect to see:

- From one month onwards the hips and knees will be flexed and the infant will need the support of your hands to sit upright. The infant should be able to keep his/her head upright.

- By 6 months old infants should be able to sit and prop themselves on their arms while keeping their head upright. Weight taken through buttocks, legs and hands. They will probably be unsteady with a tendency to fall.

The following are a cause for concern if observed during supported sitting:

- Unable to hold head steady when supported at the trunk by 6 weeks old.
- Unable to prop self on their arms while keeping head upright by 6 months old
- Very bent legs in sitting, that are not in contact with the mat by 6 months old
- Marked postural asymmetry

If you see any of these, be vigilant for signs of motor difficulties during the other TEDEI steps.

REMEMBER: independent, sustained sitting is not usually achieved until around 8 months of age.

It is a cause for concern if an infant cannot sit unsupported by 9 months of age.

Notes:

© 2019 Newcastle University

STEP 4 SUPPORT IN STANDING

Lift the infant to stand and observe posture and movement.

What you should expect to see:

- From birth (term age), infants they should take some weight through their legs, even if just intermittently.
- Head held upright and hips behind shoulders from 3 - 4 months of age

If you are not seeing any of these key things at these time points you should **CHECK AGAIN.** Remember to consider your observations along with information gained from all of the TEDEI steps. You should **REFER ON** if you have additional concerns or have had previous concerns.

The following are a cause for concern if observed during supported standing:

- Unable to keep head upright
- No weight taken through legs
- Pronounced standing on toes / tiptoes
- Marked asymmetry

If you see any of these, be vigilant for signs of motor difficulties during the other TEDEI steps.

Notes:

© 2019 Newcastle University

STEP 5 PRONE LYING

Gently place the infant on their front on the mat. Observe posture and movement.

What you should expect to see:

By 3 months:

- able to lift head to 45 degrees but turned to one side
- elbows behind shoulders

By 4 months:

- able to lift head to 45 degrees in midline
- elbows behind shoulders
- weight taken through hands, forearms and chest

By 6 months

- able to lift head beyond 45 degrees
- elbows in front of shoulders

The following are a cause for concern if observed during prone lying:

- Delay in achieving any of the above
- Unable to lift head away from surface by 2 months
- Excessive head lift and back arching (look out for other signs of high muscle tone)
- Marked asymmetry (look out for other signs of asymmetry)

If you see any of these, be vigilant for signs of motor difficulties during the other TEDEI steps.

Notes:

© 2019 Newcastle University

STEP 6 FEEL MUSCLE TONE

Turn infant from prone to supine.

Now passively move the limbs to feel muscle tone.

View the video within TEDEI to see how to do this step. If you are unsure how to do this we suggest you contact a colleague in your area who is trained and experienced in infant movement assessment and ask them to go through this step with you.

The more you handle infants, the more aware you will become of how typically developing infants feel and move, which will help you recognise when an infant may be exhibiting movement difficulties or have unusually high or low muscle tone.

1. Begin by gently holding the infant's legs below the knees and move the legs in different directions
   - Can you feel any stiffness at the hips, knees or ankles?
   - Are the legs extremely easy to move indicating very low muscle tone?
   - Do the legs feel different when you move them?
2. Now gently hold the infant's arms below the elbows and move the arms in different directions as shown here.
   - Can you feel any stiffness at the shoulders, elbows, forearms or wrists?
   - Are the hands tightly clenched?
   - Pay particular attention to the thumbs.
   - Is it difficult to move one or both thumbs out from the palm?
   - Are the arms extremely easy to move indicating very low muscle tone?
   - Do the arms feel different when you move them?
   - Can you feel any differences between each side of the body?
   - Do the legs feel stiffer than the arms or vice versa?
3. Very gently turn the infant's head to each side to check for any tightness or movement restriction in the neck.

© 2019 Newcastle University

The following are a cause for concern if felt:

- Noticeably increased muscle tone
- Very low tone or 'floppiness'
- Any other worrying findings e.g. possible dislocated hip, torticollis, should be referred on as per your usual local guidance.

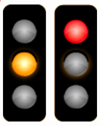

If you think you have felt something of concern then you should **CHECK AGAIN.**

Remember to consider your observations along with information gained from all of the TEDEI steps. You should **REFER ON** if you have additional concerns or have had previous concerns.

If you see any of these, be vigilant for signs of motor difficulties during the other TEDEI steps.

Notes:

© 2019 Newcastle University

Step 7 Manual Ability and Vision

1. Place infant in a supported seated position, preferably in a supportive baby chair, or otherwise held well supported in an adult's arms.

1. If the trunk is well supported, the infant will have much greater control over hand and arm movements making it much easier to reach for toys. Make sure they are able to move both arms freely and that they are not leaning on one or both arms.
2. You may use dummies or pacifiers with infants seated in baby chairs when observing fine motor play. These may help infants to be more settled and willing to handle the toys. They are also much less likely to take the toys to their mouths, therefore giving you a clearer picture of their fine motor skills.
3. Up until around 3 months old infants do not usually reach out to grasp toys. However they should look at toys, follow them with their eyes and may even move their arms towards toys (pre-reaching).

Key factors which influence the development of hand function:

1. Vision
2. Cognition
3. Posture - can the infant easily move both arms, or is their posture making this difficult?
4. Gravity
5. Environmental distractions, such as TV
6. The availability of objects and/or toys of interest and opportunities for play
7. Fatigue - young infants tire easily so watch out for signs e.g. infant becomes upset or disinterested

Birth to 3 months

Key skills to look for :

Hand Function:

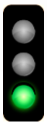

- Holds on to a toy that is placed in the hand
- Hand posture is similar on both sides
- Hands may rest in a loosely closed position

Vision:

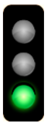

- Able to fix on and follow a toy moved slowly from side to midline
- Very interested in faces
- From 3 months the infant's eyes should be able to follow a toy moved side to side and up and down.

 © 2019 Newcastle University

At 4 months

Key manual skills to look for:

- Beginning to reach for and attempting to grasp toys of interest which are easy to get hold of
- Beginning to reach against gravity
- Will hold a toy and look at it at the same time
- Hands open readily and are not kept in fist
- Thumb/s are not kept against or within palm/s
- Can open hands to release toys
- No sign of a preferred hand
- Very visually interested in toys

REFER ON if the infant's hands are kept in a fist beyond 4 months of age

CHECK AGAIN if you are not seeing the remaining key manual skills at 4 months of age

By 6 months

Key manual skills to look for:

- Able to grasp and hold on to small toys
- Will hold some toys with both hands
- Thumbs are actively used in grasp
- Thumb/s are not kept against or within palm/s
- Can reach upwards against gravity for toys
- Turns palms of both hands face up during play (this movement is called supination)
- No sign of a preferred hand
- Very visually interested in toys

[redacted]

If you are not seeing any of these key things at these time points you should **CHECK AGAIN.** Remember to consider your observations along with information gained from the preceding TEDEI steps. You should **REFER ON** if you have additional concerns or have had previous concerns.

© 2019 Newcastle University

Notes:

© 2019 Newcastle University

TEDEI Reading List

Child Development Resources

Book: Mary Sheridan's From Birth to Five Years: Children's Developmental Progress

Authors ISBN: Ajay Sharma, Helen Cockerill
Publisher: Routledge
Paperback ISBN: 9780415833547
Hardcover ISBN: 9780415833530
eBook (VitalSource): 9780203494561

Website: Pathways

Pathways.org is a not-for-profit organization providing free child development information to empower parents and health professionals. https://pathways.org/

Some Relevant Assessments:

Book: Prechtl's General Movements Assessment

See: https://www.wiley.com/en-gb/Prechtl%27s+Method+on+the+Qualitative+Assessment+of+General+Movements+in+Preterm%2C+Term+and+Young+Infants-p-9781898683629
Course: Prechtl's General Movements Assessment visit: http://general-movements-trust.info/

Course: Hand Assessment for Infants

The Hand Assessment for Infants (HAI) is an assessment for infants between 3-12 months who have clinical signs of a unilateral cerebral palsy. Infants participate in a 10 – 15 minute play session, which is videoed and scored later. Scoring Both hands are scored independently, as well as together. The HAI can only be used by certified trainers who have attended the HAI course and then completed the post course certification procedure in three months.

http://www.ahanetwork.se

Book: Motor Assessment of the Developing Infant

Authors: Martha Piper, Johanna Darrah
Hardcover ISBN: 9780721643076
Publisher: Saunders

Assessment: Bayley Scales of Infant and Toddler Development, Third Edition (Bayley-III)

https://www.pearsonclinical.co.uk/Psychology/ChildCognitionNeuropsychologyandLanguage/ChildGeneralAbilities/BayleyScalesofInfantandToddlerDevelopmentThirdEdition(Bayley-III)/BayleyScalesofInfantandToddlerDevelopmentThirdEdition(Bayley-III).aspx

Bayley III training course also available.

e-Learning Course: Recognising neuromuscular disorders

This provides a practical approach to recognising neuromuscular disorders in children aged 0 - 5 years.

https://www.rcpch.ac.uk/resources/recognising-neuromuscular-disorders-online-learning

Other Useful Resources:

Website: EI SMART

EI SMART aims to support therapists' and families' access to effective early intervention for babies with developmental concerns
by promoting an evidence based, multi-disciplinary approach. http://www.eismart.co.uk/

© 2019 Newcastle University

Case example of what to do after identifying movement difficulties with an infant

Background:

You are a GP and have seen Scott today. Scott is just over 3 months old and his parents have no concerns about him. He is their only child. However, when they brought him for his 3 month immunisations the health visitor had been a bit concerned about his development, so you saw him a few days later for a more detailed review.

Your observations at first appointment:

While Scott was lying on his back you noticed the following:

- His trunk remained in contact with the mat throughout
- He had very little trunk rotational movement
- His hips were abducted and externally rotated
- His legs didn't move much at all
- His arms were always resting on the mat; he did not lift them or bring them to midline
- His hands were open a lot of the time
- He showed lots of visual interest, and moved his head easily to look around the room

Example of what you could say to parents after observing Scott:

"Scott is very interested in his surroundings which is really good, and he is opening his hands up very nicely too. Today I have noticed that Scott keeps his body, arms and legs resting on the mat a lot of the time. I haven't seen Scott lifting his legs off the mat, or bringing his hands together. Do you ever see him doing these things? Could you watch out for them? I would like to see him again in a couple of weeks, to check how this is progressing if that's ok?"

See / feel it once - go back and check again

Review Appointment:

You return to see Scott three weeks later. You complete all the 7 TEDEI steps with Scott and have ongoing concerns about his development. You discuss these with parents and explain that you are now going to make a referral to the community paediatrician.

See / feel it twice - refer on

© 2019 Newcastle University

Example of how you could write Scott's referral letter:

Dear Dr Jones

Re: (Scott's details)

I would be grateful if you could review this infant who is 3.5 months old. I have reviewed him twice and I am concerned about his motor development. He has reduced spontaneous movements, and lacks antigravity movements. On pulling to sit, his elbows are straight i.e. he does not assist. He also has poor head control. I don't see his hands coming to the midline, which I would expect at his age. On a positive note, he is a sociable baby who is displaying appropriate visual interest, and does not appear unwell in himself.

There is no family history of note.

I have discussed my concerns with Scott's parents, who are happy for this referral to be made.

Many thanks for your help

Yours sincerely

Dr Goodly (GP)

Notes:

© 2019 Newcastle University

Index of Video Clips

Can't add this properly until online course is finalised

© 2019 Newcastle University

© 2019 Newcastle University

Notes

© 2019 Newcastle University

Notes

© 2019 Newcastle University

Notes

© 2019 Newcastle University

Notes

© 2019 Newcastle University
