# Supplementary material for: Evaluation of the Training in Early Detection for Early Intervention (TEDEI) e-learning course using Kirkpatrick’s method
Source: BMC Med Educ. 2023 Feb 27;23:129. doi: 10.1186/s12909-023-04113-7 (PMC9968638; doi:10.1186/s12909-023-04113-7)
Supplement: Supplementary file 2 — Additional file 2: Table S1. Coding structure. [file 12909_2023_4113_MOESM2_ESM.docx]

| About the Participant | Content | | | Technical Aspects | | Impact | | |
| --- | --- | --- | --- | --- | --- | --- | --- | --- |
|  | Specific course components | Clinical Learning Points | Content quality | Media as Facilitators of Learning | Structure | Impact On Self | Impact On Practice | Fit for purpose |
|  |  |  |  |  |  |  |  |  |
| Profession | Handbook | 7 Steps Assessment | Course length | Use of Multimedia | Navigation | Confidence in referring on and when to do so | Examples of application to job role | Who can benefit and why |
| Work Setting | Case Example (written) | Traffic Lights approach | Clarity | Narration | Access Issues | Development of confidence | Working with parents | General Comments |
| Years Of Experience | Quizzes | Corrected Gestational Age | Content Specificity | Video Examples Content | Visual Design | Improving Knowledge | Screening tool | Cost/Value for Money |
| Assessment Knowledge/Experience | References | Working with Parents | Content flow | Transcripts | Refer back to content (after completion) | Refreshing Knowledge | Credence/Use as Evidence to support clinical actions | Filling a gap |
| How found out about course | Referral Letter |  |  | Video Examples Quality | Dip in and out (during) | Threat to job role if others are upskilled in this area | Training for Tele-health assessment during COVID | Online versus face to face |
| Reasons for taking course/Motivation |  |  |  |  |  | Reassurance regarding knowledge/skills | Referring Sooner |  |
| Who Paid |  |  |  |  |  |  | Structured assessment technique |  |
|  |  |  |  |  |  |  |  |  |
